# Real-World Analysis of Patients With C3 Glomerulopathy in the United States

**DOI:** 10.1016/j.ekir.2026.103773

**Published:** 2026-01-14

**Authors:** Briana C. Ndife, Carolina Aldworth, Jennifer Nguyen, Irina Pivneva, Marie Louise Edwards, Annika Anderson, James Signorovitch, Pietro A. Canetta

**Affiliations:** 1 Health Economics and Outcomes Research, Novartis Pharmaceuticals Corporation, East Hanover, New Jersey, USA; 2 Analysis Group, Boston, Massachusetts, USA; 3 Columbia University Irving Medical Center, New York, New York, USA

**Keywords:** alternative complement pathway, C3 glomerulopathy, chronic kidney disease, glomerulonephritis, kidney diseases, kidney transplantation

## Abstract

**Introduction:**

C3 glomerulopathy (C3G) is a rare kidney disease caused by alternative pathway (AP) overactivation. This analysis describes demographic and clinical characteristics of patients with C3G in the United States (US) and evaluates chronic kidney disease (CKD) stage progression.

**Methods:**

This retrospective cohort study used electronic health records (EHRs) to identify patients aged ≥ 12 years with a C3G diagnosis (index) and with continuous clinical activity ≥ 12 months before (baseline) and ≥ 6 months after index (follow-up; until death or data end). Patients were stratified by prior kidney transplant status, CKD stage progression during follow-up, and baseline serum C3 level. CKD stage progression was assessed in patients with CKD stages 1 to 4 (at index) and adequate data to assess progression during follow-up.

**Results:**

Overall, 260 patients with C3G were identified: 51.9% were female; mean (SD) age was 47.8 (20.6) years; median follow-up was 2.1 years. Of those assessed, most had CKD stage ≥ 3 (56.6%) and proteinuria (54.4%) at index. Posttransplant recurrent C3G was reported in 27 patients (10.4%). Of those assessed for CKD stage progression, 102 (59.0%) and 45 (26.0%) progressed to a higher CKD stage or CKD stage 5 or kidney failure, respectively. Median time to progression of CKD stage or to stage 5 or kidney failure was 14.9 months and not estimable (NE), respectively.

**Conclusion:**

In a real-world US population of patients with C3G, we identified a population with both advanced kidney disease around the time of diagnosis and high rates of CKD stage progression, highlighting the need to utilize novel treatments to improve patient outcomes.

## Graphical abstract

**Figure:**
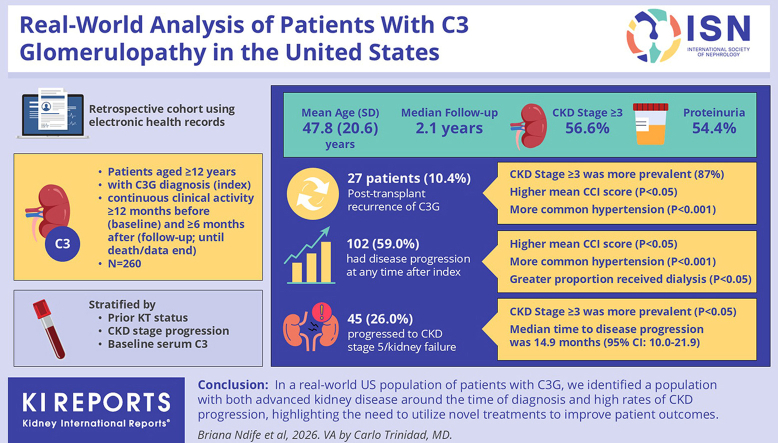

C3G is a chronic, rare kidney disease with an estimated incidence of 1 to 3 cases per million people in the US.^1^^,^^2^ C3G encompasses 2 major subtypes of kidney disease, namely dense deposit disease and C3 glomerulonephritis. C3G is characterized by the accumulation of C3 in the glomeruli, caused by overactivation of the AP.^3^ Normally, the AP is both persistently active at a low level and tightly controlled; however, AP overactivation can result from genetic or acquired factors, such as autoantibodies to complement proteins.^1^^,^^4^ C3 glomerular deposition triggers inflammation, glomerular injury, and progressive kidney disease.^1^^,^^3^^,^^4^

C3G is often observed in children and young adults, and has a variable clinical presentation.^2^^,^^5^ Patients commonly present with proteinuria and hematuria; however, immunofluorescence staining and kidney biopsy are required for C3G diagnosis.^1^^,^^6^ Some patients with C3G may have stable kidney function for several years despite persistent proteinuria. Others may have rapid fluctuations in proteinuria, leading to episodes of acute kidney deterioration, often in the absence of obvious triggers.^7^ The prognosis for patients with C3G is poor: up to 50% of adults develop kidney failure within 10 years of diagnosis, requiring dialysis or transplant.^1^^,^^5^^,^^7^^,^^8^ Furthermore, the disease frequently recurs after transplant; posttransplant recurrent C3G is the main cause of graft failure.^5^^,^^7^^,^^9^, ^10^, ^11^

There were no validated targeted treatment strategies for C3G until the US Food and Drug Administration approval of iptacopan in March 2025.^12^^,^^13^ Iptacopan is an oral, first-in-class, specific inhibitor of factor B, which is approved to treat adult patients with C3G to reduce proteinuria.^13^^,^^14^ More recently, pegcetacoplan, a C3 and C3b inhibitor, received US Food and Drug Administration approval for the treatment of C3G and primary immune complex membranoproliferative glomerulonephritis.^15^ However, the current Kidney Disease: Improving Global Outcomes guidelines were published in 2021 and recommend the use of supportive care (including angiotensin-converting enzyme inhibitors or angiotensin II receptor blockers) and immunosuppressive agents (including corticosteroids and mycophenolate mofetil) for patients with moderate-to-severe disease, based on expert opinion and limited data from retrospective studies.^12^ In addition, the use of the anti-C5 monoclonal antibody, eculizumab, may be considered in patients who fail to respond to other therapies, though outcomes have been variable.^12^

There are significant gaps in our understanding of the natural history of C3G, because of the rarity of the disease and the limited availability of cohort studies.^1^^,^^2^^,^^16^ To date, most C3G cohort studies in children and adults have been performed in Europe and Asia,^5^^,^^10^^,^^17^, ^18^, ^19^, ^20^, ^21^, ^22^, ^23^ with only 2 studies in the USA.^2^^,^^24^ Both US cohort studies were single-center studies (albeit with large academic referral centers) so may not accurately represent a diverse US population. This analysis describes the demographics and clinical characteristics of patients with C3G, and the timing of CKD stage progression, in real-world US clinical practice, using EHR data from a large, integrated clinical delivery network.

## Methods

### Study Design

This study was performed in a retrospective cohort of US patients within the Optum deidentified EHR data set (Optum EHR)^25^ who received a C3G diagnosis between January 2015 and June 2022 (data accessed 2023) (Supplementary Figure S1). Optum EHR is a longitudinal EHR repository derived from dozens of health care provider organizations in the US. Administrative medical data are obtained from both inpatient and ambulatory EHRs, practice management systems, and other internal systems, and are processed, normalized, and standardized across the continuum of care from both acute inpatient stays and outpatient visits. The data are statistically deidentified under the Health Insurance Portability and Accountability Act Privacy Rule’s Expert Determination method and managed according to Optum customer data use agreements.

The index date was defined as the date of first C3G diagnosis. Data on patient demographics, clinical characteristics, diagnostic journey, CKD stage, and laboratory values were collected during baseline (January 2013–December 2021), defined as the period of ≥ 12 months prior to index. Data on clinical outcomes, clinical characteristics, CKD stage progression, and laboratory values were collected during follow-up (January 2015–June 2022), defined as the period of ≥ 6 months from index until the earliest of data end, patient death, or end of continuous clinical activity.

### Participants

Patients were included if they had ≥ 1 diagnosis code for C3G (International Classification of Diseases, 10th Revision, Clinical Modification [ICD-10-CM] or Systemized Nomenclature of Medicine Clinical Terms codes) (Supplementary Table S1), were aged ≥ 12 years at index, and had continuous clinical activity ≥ 12 months before and ≥ 6 months after index. A sensitivity analysis was also conducted among the subset of patients with ≥ 2 C3G diagnosis codes on distinct dates (Supplementary Sensitivity Analysis). Patients with monoclonal gammopathy of undetermined significance, identified by ICD-9-CM, ICD-10-CM, and Systemized Nomenclature of Medicine Clinical Terms codes, were excluded. All patients who met the inclusion criteria without meeting any exclusion criteria were included. Patients were stratified into groups reflective of clinically relevant features, including the following: (i) kidney transplant status at index date (C3G in the native kidney, or posttransplant recurrent C3G, defined as documentation of a kidney transplant during baseline); (ii) C3 level, as assessed using data closest to index (decreased [< 77 mg/dl] or normal [≥ 77 to < 201 mg/dl]); and (iii) CKD stage progression status (CKD stage progressors or nonprogressors, as assessed during follow-up). CKD stage at index date was defined using the estimated glomerular filtration rate (eGFR) value closest to index (± 1 month). CKD stage was defined as stage 1 (eGFR ≥ 90 ml/min per 1.73 m^2^), stage 2 (eGFR: 60–89 ml/min per 1.73 m^2^), stage 3a (eGFR: 45–59 ml/min per 1.73 m^2^), stage 3b (eGFR: 30–44 ml/min per 1.73 m^2^), stage 4 (eGFR: 15–29 ml/min per 1.73 m^2^), and stage 5 (eGFR < 15 ml/min per 1.73 m^2^ or on dialysis). If eGFR data were unavailable at index, CKD stage was defined using the closest CKD stage diagnosis code (Supplementary Table S2). If a patient had a dialysis procedure code during baseline, their CKD stage at index was defined as stage 5 or kidney failure. CKD stage progression was assessed between index date and follow-up time points in all patients with CKD stages 1 to 4 (and no dialysis) at index who had adequate data to assess progression (based on laboratory values, diagnosis codes, or dialysis procedure codes). For the progression-free survival analysis, patients with CKD stage 5 or kidney failure, defined by a value before or at index, were excluded. Patients with CKD stage 5 or kidney failure, defined by a value within 1 month after index, were included in the analyses, and considered progressed on the date of the procedure, laboratory evaluation, or diagnosis data that defined the CKD stage at index. Patients reaching a higher CKD stage (derived using a combination of diagnosis or procedure codes, plus laboratory values) during follow-up compared with index were considered to have disease progression. eGFR slopes were calculated for each patient as the change in eGFR/yr from baseline to the end of follow-up. Patients were required to have ≥ 1 eGFR value in each of the baseline and follow-up periods, and a difference of ≥ 6 months between their last baseline eGFR value and last follow-up eGFR value. The eGFR slope for each patient was calculated with a linear regression fit to the eGFR value closest to the end of baseline, and all available follow-up eGFR data. To measure change in eGFR from baseline, the intercept of linear regression for each patient was fixed to the baseline eGFR value. Proteinuria slopes were calculated for each patient as the change in proteinuria/mo from baseline to the end of follow-up. Patients were required to have ≥ 1 urine protein-to-creatinine ratio (UPCR) in each of the baseline and follow-up periods, and a difference of ≥ 1 month between their last baseline UPCR and last follow-up UPCR. The proteinuria slope for each patient was calculated with a linear regression fit to the UPCR closest to the end of baseline and all available follow-up UPCR data. To measure change in proteinuria from baseline, the intercept of linear regression for each patient was fixed to the baseline UPCR.

### Objectives

Objectives of this study included the following: (i) to describe the demographic and clinical characteristics of patients with C3G at index, (ii) to estimate the proportion of patients with C3G at high risk of progression despite optimized supportive care, (iii) to estimate the proportion of patients with C3G who progressed to increased CKD stage and/or kidney failure, and (iv) to estimate the time from index to CKD stage progression and/or kidney failure.

### Statistical Analysis

Results were analyzed using R and SAS Enterprise Guide 7.1 (SAS Institute, Inc., Cary, NC) and were reported descriptively. Continuous variables were summarized as mean and SD, with median and interquartile range (IQR) or range reported for some variables. Categorical variables were summarized by counts and percentages. Statistical comparisons between patient demographics and clinical characteristics were performed using Chi-square test or Fisher exact test (for variables with any expected value[s] < 5) for categorical variables and *t* tests for continuous variables. Time to CKD stage progression and time to CKD stage 5 or kidney failure were estimated using Kaplan-Meier analyses and reported as medians and 95% confidence intervals (CIs). eGFR and proteinuria slope estimates were summarized as a descriptive summary of individual patients’ slope results. The relationship between transplant status at index and baseline C3 level versus progression to higher CKD stage or CKD stage 5 were examined using a multivariable Cox proportional hazards model and reported as hazard ratios and 95% CIs. The models for transplant status were adjusted for age at index, sex (sex was determined as it was reported in the Optum EHR data, which represents how health care providers recorded patient information), CKD stage at index, baseline Charlson Comorbidity Index (CCI) score, baseline supportive therapy, and baseline immunosuppressive agents. The models for baseline C3 level were adjusted for age at index, sex, CKD stage at index, baseline body mass index, and baseline supportive therapy.

### Conduct and Ethics

This analysis was conducted in accordance with the ethical principles of the Declaration of Helsinki and Strengthening the Reporting of Observational Studies in Epidemiology (STROBE) guidelines. All data used in this analysis were anonymized.

## Results

### Baseline Patient Demographics, Clinical Characteristics, and Treatments

**Figure 1:**
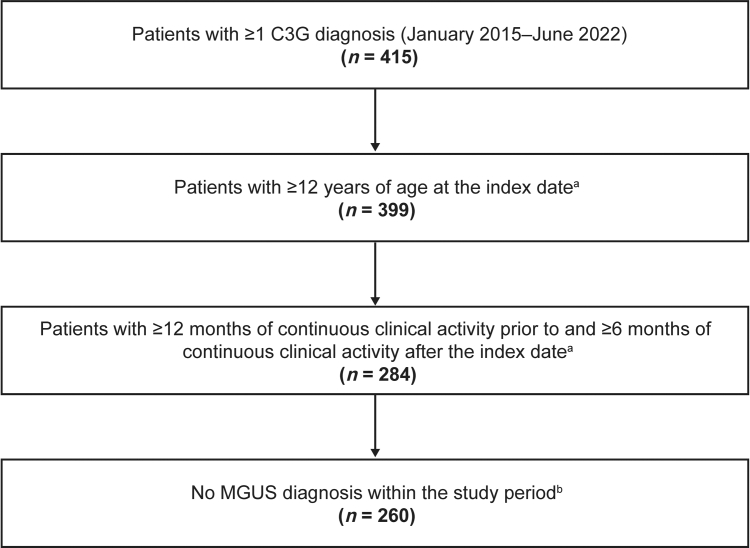
Patient flow diagram. C3G, C3 glomerulopathy; MGUS, monoclonal gammopathy of undetermined significance. ^a^Index was defined as the date of the first C3G diagnosis. ^b^The study period includes the baseline (≥ 12 months before index) and follow-up (≥ 6 months after and including index) periods.

The database contained records from > 102 million patients, 415 of whom had ≥ 1 C3G diagnosis, with 260 meeting all inclusion criteria (Figure 1). Demographic and clinical characteristics for the overall population, stratified by kidney transplant status, C3 level (low vs. normal), and CKD stage progression status, are summarized in Table 1.^26^^,^^27^ Most patients were White (78.1%), 51.9% were female, and mean (SD) age was 47.8 (20.6) years. Median follow-up time was 2.1 years. Of the 205 patients with available CKD stage data at index date, 56.6% had CKD stage ≥ 3. Based on data closest to index, 58 of 91 patients (63.7%) with available data had normal C3 levels. Of patients with proteinuria values at baseline (*n* = 114, 43.8%), mean (SD) UPCR was 2.8 (3.7) g/g, and 62 (54.4%) patients had a proteinuria level ≥ 1 g/g. Hematuria values were reported for 92 patients (35.4%), and mean (SD) number of red blood cells per high power field was 31.5 (48.7) (Table 1). Of patients with eGFR assessed (*n* = 218, 83.8%), mean (SD) eGFR was 61.6 (37.5) ml/min per 1.73 m^2^. The mean (SD) CCI score was 2.1 (2.5), and hypertension was the most common C3G-related comorbidity (62.7%) (Supplementary Table S3; diagnosis codes are presented in Supplementary Tables S4 and S5). The most common C3G-related treatments before index were cardiovascular-related (62.3%) and supportive therapies (angiotensin-converting enzyme inhibitor and angiotensin II receptor blockers, 54.6%) (Table 1; a full list of treatments are presented in Supplementary Table S6).

**Table 1:** Patient demographics and clinical characteristics for the overall population and stratified by transplant, C3 level, and progression status

Characteristic	Overall (*N* = 260)	Kidney status at index (*N* = 260)		*P*-value	Status by C3 level^a^*(n* = 91)		*P*-value	Patients with CKD stage assessed during follow-up^b^(*n* = 173)		
		C3G in the native kidney (*n* = 233)	Posttransplant recurrent C3G (*n* = 27)		Decreased (*n* = 33)	Normal (*n* = 58)		Non-progressors (*n* = 71)	CKD stage progressors (*n* = 102)	*P*-value
Age at index, yr										
Mean ± SD	47.8 ± 20.6	48.1 ± 20.9	44.6 ± 18.5	0.40	33.3 ± 20.6	44.4 ± 20.9	< 0.05^c^	47.5 ± 19.9	52.2 ± 20.3	0.14
Sex, *n* (%)										
Female	135 (51.9)	124 (53.2)	11 (40.7)	0.31	17 (51.5)	30 (51.7)	1.00	36 (50.7)	52 (51.0)	1.00
Male	125 (48.1)	109 (46.8)	16 (59.3)		16 (48.5)	28 (48.3)		35 (49.3)	50 (49.0)	
Race, *n* (%)										
African American	26 (10.0)	23 (9.9)	3 (11.1)	0.62	3 (9.1)	5 (8.6)	0.97	3 (4.2)	13 (12.7)	< 0.05^c^
Asian	6 (2.3)	6 (2.6)	0		1 (3.0)	3 (5.2)		4 (5.6)	1 (1.0)	
White	203 (78.1)	183 (78.5)	20 (74.1)		24 (72.7)	43 (74.1)		54 (76.1)	81 (79.4)	
Other/unknown	25 (9.6)	21 (9.0)	4 (14.8)		5 (15.2)	7 (12.1)		10 (14.1)	7 (6.9)	
CKD stage within 1 mo of index,^d^*n* (%)										
CKD stage assessed	205 (78.8)	182 (78.1)	23 (85.2)	0.47	26 (78.8)	55 (94.8)	< 0.05^c^	71 (100.0)	102 (100.0)	-
Stage 1	40 (19.5)	39 (21.4)	1 (4.3)	< 0.01^c^	9 (34.6)	6 (10.9)	< 0.05^c^	22 (31.0)	18 (17.6)	0.05
Stage 2	49 (23.9)	47 (25.8)	2 (8.7)		2 (7.7)	10 (18.2)		23 (32.4)	26 (25.5)	
Stage 3	40 (19.5)	33 (18.1)	7 (30.4)		4 (15.4)	15 (27.3)		14 (19.7)	26 (25.5)	
Stage 4	31 (15.1)	29 (15.9)	2 (8.7)		5 (19.2)	9 (16.4)		12 (16.9)	19 (18.6)	
Stage 5 or kidney failure	45 (22.0)	34 (18.7)	11 (47.8)		6 (23.1)	15 (27.3)		0	13 (12.7)	
Treatments during baseline, *n* (%)										
Cardiovascular-related^e^	162 (62.3)	138 (59.2)	24 (88.9)	< 0.01^c^	21 (63.6)	44 (75.9)	0.32	37 (52.1)	83 (81.4)	< 0.001^c^
Supportive therapy	142 (54.6)	126 (54.1)	16 (59.3)	0.76	25 (75.8)	40 (69.0)	0.65	36 (50.7)	69 (67.6)	< 0.05^c^
ACEi	106 (40.8)	94 (40.3)	12 (44.4)	0.84	18 (54.5)	31 (53.4)	1.00	25 (32.2)	53 (52.0)	< 0.05^c^
ARBs	65 (25.0)	56 (24.0)	9 (33.3)	0.41	11 (33.3)	22 (37.9)	0.83	16 (22.5)	32 (31.4)	0.27
Corticosteroid (oral/i.v.)	141 (54.2)	118 (50.6)	23 (85.2)	< 0.001^c^	23 (69.7)	38 (65.5)	0.86	31 (43.7)	70 (68.6)	< 0.01^c^
Other immunosuppressive agents	59 (22.7)	36 (15.5)	23 (85.2)	< 0.001^c^	14 (42.4)	26 (44.8)	1.00	13 (18.3)	25 (24.5)	0.43
Mycophenolate mofetil	38 (14.6)	19 (8.2)	19 (70.4)	< 0.001^c^	10 (30.3)	18 (31.0)	1.00	7 (9.9)	16 (15.7)	0.38
Tacrolimus	28 (10.8)	7 (3.0)	21 (77.8)	< 0.001^c^	6 (18.2)	12 (20.7)	0.99	3 (4.2)	15 (14.7)	< 0.05^c^
Eculizumab	7 (2.7)	3 (1.3)	4 (14.8)	< 0.01^c^	2 (6.1)	4 (6.9)	1.00	2 (2.8)	2 (2.0)	1.00
eGFR (ml/min per 1.73 m^2^),^a^^,^^f^*n* (%)										
eGFR measured	218 (83.8)	193 (82.8)	25 (92.6)	0.27	31 (93.9)	56 (96.6)	0.62	68 (95.8)	92 (90.2)	0.24
eGFR mean ± SD	61.6 ± 37.5	64.8 ± 37.7	37.4 ± 25.1	< 0.001^c^	60.4 ± 44.5	44.7 ± 31.9	0.06	78.1 ± 35.2	53.2 ± 31.8	< 0.001^c^
Proteinuria status,^a^^,^^g^*n* (%)										
Proteinuria status assessed	114 (43.8)	97 (41.6)	17 (63.0)	0.06	27 (81.8)	49 (84.5)	0.97	33 (46.5)	54 (52.9)	0.50
Normal (< 0.2 g/g)	16 (14.0)	10 (10.3)	6 (35.3)	< 0.05^c^	2 (7.4)	6 (12.2)	0.43	6 (18.2)	10 (18.5)	0.46
Subnephrotic (≥ 0.2 to < 3.5 g/g)	70 (61.4)	61 (62.9)	9 (52.9)		15 (55.6)	32 (65.3)		21 (63.6)	28 (51.9)	
Nephrotic (≥ 3.5 g/g)	28 (24.6)	26 (26.8)	2 (11.8)		10 (37.0)	11 (22.4)		6 (18.2)	16 (29.6)	
Hematuria status,^a^^,^^h^ (RBCs/HPF), *n* (%)										
Hematuria assessed	92 (35.4)	77 (33.0)	15 (55.6)	< 0.05^c^	20 (60.6)	32 (55.2)	0.78	30 (42.3)	43 (42.2)	1.00
Normal (< 3)	30 (32.6)	25 (32.5)	5 (33.3)	1.00	1 (5.0)	11 (34.4)	< 0.05^c^	12 (40.0)	14 (32.6)	0.69
Microscopic hematuria (≥ 3)	62 (67.4)	52 (67.5)	10 (66.7)		19 (95.0)	21 (65.6)		18 (60.0)	29 (67.4)	
C3G-related procedures, *n* (%)										
Kidney biopsy	53 (20.4)	41 (17.6)	12 (44.4)	< 0.01^c^	13 (39.4)	29 (50.0)	0.45	11 (15.5)	29 (28.4)	0.07
Kidney transplant^i^	27 (10.4)	0	27 (100.0)	< 0.001^c^	3 (9.1)	13 (22.4)	0.15	2 (2.8)	11 (10.8)	0.08
Hemodialysis	23 (8.8)	17 (7.3)	6 (22.2)	< 0.05^c^	7 (21.2)	7 (12.1)	0.39	1 (1.4)	10 (9.8)	< 0.05^c^

ACEi, angiotensin-converting enzyme inhibitors; ARB, angiotensin II receptor blockers; C3, complement component 3; C3G, C3 glomerulopathy; CKD, chronic kidney disease; CKD-EPI, CKD Epidemiology Collaboration; eGFR, estimated glomerular filtration rate; HPF, high power field; RBC, red blood cell; UPCR, urine protein-to-creatinine ratio.

a Assessed using data closest to index.

b Patients with a lower CKD stage at index than at the follow-up timepoint were considered progressed.

c Indicates *P* < 0.05.

d CKD stage was defined using the eGFR value closest to index. eGFR laboratory values were either calculated using the CKD-EPI creatinine equation (2021) for adult patients (aged ≥ 18 years) or reported by Optum (Schwartz formula) for pediatric patients (aged < 18 years). If eGFR laboratory data were not available within the specified period, CKD stage was defined using the CKD stage diagnosis code closest to index. If a patient had a procedure code for dialysis within the specified time period, their CKD stage for that period was defined as CKD stage 5/kidney failure.

e Cardiovascular-related treatments comprised beta blockers, diuretics, mineralocorticoid receptor antagonists, and statins.

f eGFR laboratory values were either calculated using the CKD-EPI creatinine equation (2021) for adult patients (aged ≥ 18 years) or reported by Optum (Schwartz formula) for pediatric patients (aged < 18 years).

g Proteinuria was assessed using UPCR; proteinuria status was based on the definition from Kamińska *et al.*^26^

h Hematuria was assessed using RBC count (microscopic urinalysis), and hematuria status was based on the definition from Barocas *et al.*^27^

i Kidney transplant during baseline, or diagnosis code at baseline, indicating a prior kidney transplant.

#### Patients Stratified by Posttransplant Recurrent C3G and C3G in the Native Kidney

At index, 27 patients (10.4%) had presumed posttransplant recurrent C3G, and 233 patients (89.6%) had C3G in the native kidney (Table 1). Among patients with posttransplant recurrent C3G versus those with C3G in the native kidney, CKD stage ≥ 3 was more prevalent (87.0% vs. 52.7%, of patients with available CKD stage assessment), mean CCI score was significantly higher (3.3 vs. 2.0; *P* < 0.05), and C3G-related comorbidities were more common (hypertension, 92.6% vs. 59.2%; *P* < 0.001) (Supplementary Table S3). Use of C3G-related treatments was significantly higher in patients with posttransplant recurrence than in patients with C3G in the native kidney (cardiovascular-related, 88.9% vs. 59.2% [*P* < 0.01]; corticosteroids, 85.2% vs. 50.6% [*P* < 0.001]; immunosuppressive agents, 85.2% vs. 15.5% [*P* < 0.001]).

#### Patients Stratified by Baseline C3 Level

Of the 260 included patients, 91 (35.0%) had a recorded C3 level at baseline: 58 had normal C3 levels (77 to < 201 mg/dl) and 33 had low C3 levels (< 77 mg/dl) (Table 1). For patients with normal C3 levels versus those with low C3, CKD stage ≥ 3 was more prevalent (70.9% and 57.7%, respectively), C3G-related comorbidities were more common (hypertension, 79.3% and 60.6%, respectively [*P* = 0.09]; fatigue, 51.7% and 24.2%, respectively [*P* < 0.05]), use of C3G-related treatments was similar between groups, and mean (SD) CCI score was numerically lower in patients with normal C3 than in those with decreased C3 (2.5 [2.2] and 3.0 [3.3] , respectively; *P* = 0.35) (Supplementary Table S3).

#### Patients Stratified by CKD Stage Progression

Of the 173 patients assessed for CKD stage progression, 102 (59.0%) had disease progression at any time after index, and 71 (41.0%) were classified as nonprogressors (Table 1). During baseline, disease progressors had significantly higher mean (SD) CCI score (2.7 [2.6] vs. 1.8 [2.4], *P* < 0.05) and more C3G-related comorbidities, including hypertension (79.4% vs. 53.5%, *P* < 0.001), and a greater proportion received hemodialysis (9.8% vs. 1.4%, *P* < 0.05). In addition, the use of cardiovascular-related treatments, supportive therapies, and corticosteroids was significantly higher in patients with CKD stage progression compared with nonprogressors (cardiovascular-related, 81.4% vs. 52.1% [*P* < 0.001]; supportive therapies, 67.6% vs. 50.7% [*P* < 0.05]; corticosteroids, 68.6% vs. 43.7% [*P* < 0.01]) (Table 1).

### CKD Stage Progression

**Figure 2:**
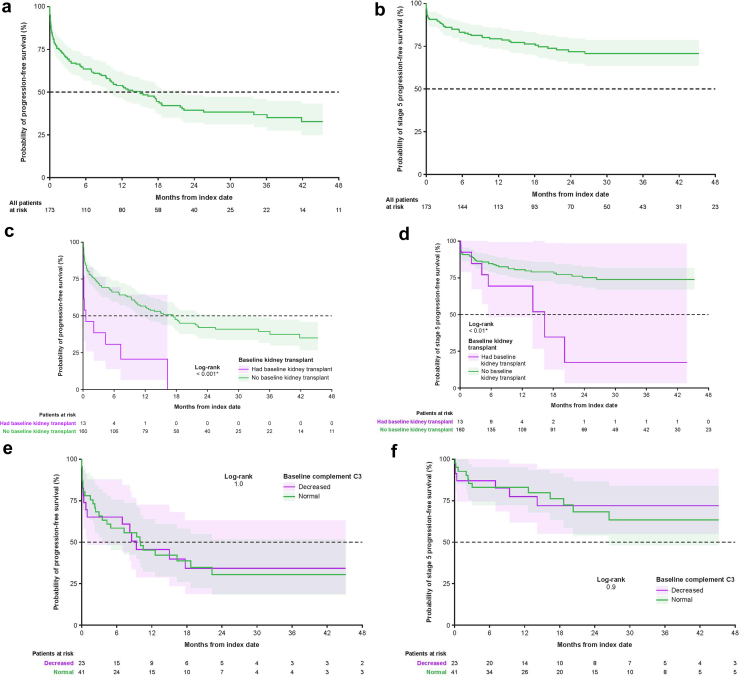
Time to chronic kidney disease stage progression and time to progression to chronic kidney disease stage 5 or kidney failure, respectively, in (a and b) the overall population (*n* = 173), (c and d) patients stratified by kidney transplant status, and (e and f) patients stratified by C3 level. ∗Indicates *P* < 0.05.

**Figure 3:**
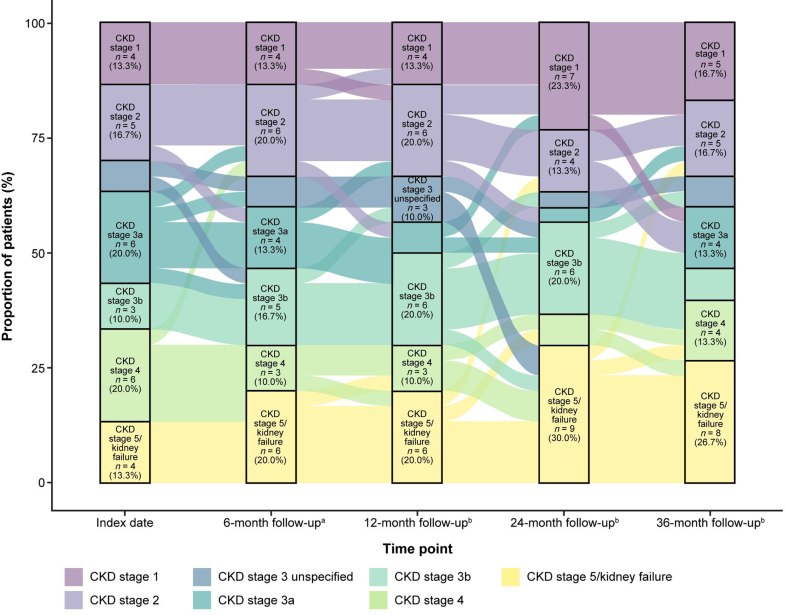
Chronic kidney disease staging over time. CKD, chronic kidney disease. Note: This Sankey diagram shows the changes in chronic kidney disease staging in patients with C3 glomerulopathy from index. Overall, 30 patients had chronic kidney disease stage assessed at all time points. ^a^6 months from index ± 1 month. ^b^12, 24, or 36 months from index ± 3 months.

Of those assessed for CKD stage progression (*n* = 173), 45 (26.0%) progressed to CKD stage 5 or kidney failure during follow-up (Table 2). At index date, relative to nonprogressors, patients who had disease progression were more likely to have CKD stage ≥ 3 (56.9% vs. 36.6%, *P* < 0.05) (Table 1). Median time to disease progression was 14.9 months (95% CI: 10.0–21.9) (Table 2). Median time to CKD stage 5 or kidney failure was not reached. CKD stage progression over time in all patients, as well as stratified by C3 level and kidney transplant status at index date, is shown in Supplementary Table S7. Time to CKD stage progression for the overall population, and stratified by C3 level and kidney transplant status, is shown in Figure 2. The patient-level journey of CKD stage progression assessed at 6-, 12-, 24-, and 36-months postindex is shown in Figure 3.

**Table 2:** Incidence and timing of CKD stage progression

CKD stage progression, *n* (%)	Patients with CKD stage progression assessed during follow-up (*N* = 173)	Kidney status at the index date (*n* = 173)		Status by C3 level (*n* = 64)	
		C3G in the native kidney (*n* = 160)	Posttransplant recurrent C3G (*n* = 13)	Decreased (*n* = 23)	Normal (*n* = 41)
Progressed CKD stage^a^	102 (59.0)	91 (56.9)	11 (84.6)	14 (60.9)	26 (63.4)
Progressed to CKD stage 5 or kidney failure	45 (26.0)	38 (23.8)	7 (53.8)	6 (26.1)	12 (29.3)
Time to CKD stage progression, mo^b^					
Median (95% CI)	14.9 (10.0–21.9)	17.3 (11.0–33.9)	0.5 (0.1–NE)	9.4 (1.0–NE)	10.0 (4.3–NE)
Progression-free rate estimate, %					
6 mos	63.6	66.3	30.8	65.2	58.5
12 mos	54.0	56.7	20.5	45.7	45.2
24 mos	39.5	42.0	NA	34.2	30.5
36 mos	36.9	39.3	NA	34.2	30.5

C3, complement component 3; C3G, C3 glomerulopathy; CI, confidence interval; CKD, chronic kidney disease; NA, not available; NE, not estimable.

a Patients with a lower CKD stage at index than at the follow-up timepoint were considered progressed.

b Time to progression was measured from the index date to the date of the first instance of progression after the index date, and the date of the laboratory values or diagnosis data that defined the CKD stage at index. Time to progression for patients with CKD stage 5/kidney failure at index defined with procedure, laboratory, or diagnosis data after index (within 1 mo of index) was measured from index to the date of the procedure, laboratory, or diagnosis data that defined the CKD stage at index.

#### Patients Stratified by Posttransplant Recurrent C3G and C3G in the Native Kidney

Of the patients assessed for CKD stage progression (*n* = 173), 13 (7.5%) had posttransplant recurrent C3G (Table 2), of whom 11 (84.6%) experienced disease progression during the observation period. Among patients with native kidneys assessed for CKD stage progression (*n* = 160), 91 (56.9%) showed disease progression. Progression to CKD stage 5 or kidney failure occurred in 7 patients (53.8%) with posttransplant recurrent C3G and 38 patients (23.8%) with C3G in the native kidney. Median time to disease progression was significantly shorter in patients with posttransplant recurrent C3G (0.5 months [95% CI: 0.1‍–‍NE]) compared with patients with C3G in the native kidney (17.3 months [95% CI: 11.0–33.9], *P* < 0.001). Patients with posttransplant recurrent C3G were 4 times more likely to have disease progression to a higher CKD stage than those with C3G in the native kidney (hazard ratio: 4.0 [95% CI: 1.70–9.46], *P* < 0.01) (Supplementary Table S7).

#### Patients Stratified by Baseline C3 Level

Of the 64 patients assessed for CKD stage progression who had a C3 level at index date, 41 (64.1%) had normal C3 levels and 23 (35.9%) had decreased C3 levels (Table 2). Disease progression occurred in 26 (63.4%) and 14 (60.9%) patients with normal and decreased C3 levels, respectively. Progression to CKD stage 5 or kidney failure occurred in 12 (29.3%) and 6 (26.1%) patients with normal and decreased C3 levels, respectively. Median time to disease progression among patients with normal and decreased C3 levels was 10.0 months (95% CI: 4.3–NE) and 9.4 months (95% CI: 1.0–NE), respectively. There was no significant difference in the likelihood of disease progression to a higher CKD stage between patients with normal and decreased C3 levels (hazard ratio: 1.25 [95% CI: 0.58–2.70], *P* = 0.56) (Supplementary Table S8). In the sensitivity analysis sample, 35 patients were assessed for CKD stage progression and had a C3 level reported at index; 22 (62.9%) had normal C3 levels and 13 (37.1%) had decreased C3 levels. Patients with decreased C3 levels were 4 times more likely to have disease progression to a higher CKD stage than those with normal C3 levels (hazard ratio: 4.05 [95% CI: 1.23‍–‍13.29], *P* < 0.05) (Supplementary Sensitivity Analysis).

### Postindex Clinical Characteristics and Treatments

Median (IQR) annual eGFR slope, calculated for each patient (*n* = 170) as the change in eGFR/yr from baseline to the end of follow-up, was −0.9 (−7.5 to 3.8) ml/min per 1.73 m^2^/yr (Table 3). Among the patients included in this analysis, the median (IQR) number of eGFR results per patient used to calculate the eGFR slope was 12.0 (5.0–29.5). Median (IQR) annual eGFR slope was more negative in patients with posttransplant recurrent C3G than in those with C3G in the native kidney (−1.6 [−9.3 to 4.6] ml/min per 1.73 m^2^/yr vs. −0.9 [‍−‍6.1 to 3.8] ml/min per 1.73 m^2^/yr); patients with decreased C3 levels versus normal C3 levels (−2.5 [−‍17.8 to 2.7] ml/min per 1.73 m^2^/yr vs. −0.6 [−8.0 to 7.1] ml/min per 1.73 m^2^/yr); and patients with CKD stage progression versus nonprogressors (−2.1 [−11.4 to 2.0] ml/min per 1.73 m^2^/yr vs. −0.7 [‍−‍3.5 to 4.3] ml/min per 1.73 m^2^/yr). Median (IQR) monthly proteinuria slope, calculated for each patient (*n* = 79) as the change in proteinuria/mo from baseline to the end of follow-up, was 0.0 (−0.1 to 0.0) g/g/mo.

**Table 3:** eGFR slope analysis for the overall population and stratified by transplant, C3 level, and progression status

eGFR slope^c^ (ml/min per 1.73 m^2^/yr)	Overall (*N* = 170)	Kidney status at the index date (*N* = 170)		Status by C3 level^a^*(n* = 71)		Patients with CKD stage assessed during follow-up^b^ (*n* = 129)	
		C3G in the native kidney (*n* = 151)	Posttransplant recurrent C3G (*n* = 19)	Decreased (*n* = 27)	Normal (*n* = 44)	Non-progressors (*n* = 45)	CKD stage progressors (*n* = 84)
Mean ± SD	0.3 ± 27.8	−0.6 ± 25.0	7.4 ± 44.9	−1.1 ± 43.4	4.5 ± 32.5	0.0 ± 11.2	−3.0 ± 30.9
Median	−0.9	−0.9	−1.6	−2.5	−0.6	−0.7	−2.1
IQR	−7.5 to 3.8	−6.1 to 3.8	−9.3 to 4.6	−17.8 to 2.7	−8.0 to 7.1	−3.5 to 4.3	−11.4 to 2.0
Range	−70.6 to 195.5	−50.3 to 195.5	−70.6 to 112.2	−48.6 to 195.5	−70.6 to 140.7	−47.5 to 22.5	−70.6 to 195.5

C3, complement component 3; C3G, C3 glomerulopathy; CKD, chronic kidney disease; CKD-EPI, Chronic Kidney Disease Epidemiology Collaboration; eGFR, estimated glomerular filtration rate; IQR, interquartile range.

a Assessed using data closest to index.

b Patients with a lower CKD stage at index than at the follow-up timepoint were considered progressed.

c eGFR laboratory values were either calculated with the CKD-EPI creatinine equation (2021) for adult patients (aged ≥18 years) or reported by Optum (Schwartz formula) for pediatric patients (aged <18 years). The eGFR slope for each patient was calculated with a linear regression fit to the baseline eGFR value closest to the end of baseline and all available follow-up eGFR data. To measure change in eGFR from baseline, the intercept of linear regression for each patient was fixed to the baseline eGFR value.

The overall change in eGFR and proteinuria for patients with baseline and follow-up assessments are shown in Table 4. eGFR and proteinuria results during follow-up for the subsets of patients with CKD stage progression at 6-, 12-, 24-, and 36-months postindex are shown in Supplementary Table S9.

**Table 4:** Change in eGFR and proteinuria

eGFR and proteinuria	Baseline	Follow-up	Change
Patients with baseline and follow-up eGFR assessment (*n* = 195)^a^^,^^b^^,^^c^			
eGFR (ml/min per 1.73 m^2^)	58.7 ± 37.1	59.0 ± 36.7	0.3 ± 26.0
Patients with baseline and follow-up proteinuria assessment (*n* = 80)^a^^,^^d^			
UPCR (g/g)	2.6 ± 3.4	2.0 ± 2.9	−0.6 ± 3.1

CKD-EPI, Chronic Kidney Disease Epidemiology Collaboration; eGFR, estimated glomerular filtration rate; IQR, interquartile range; UPCR, urine total protein-to-creatinine ratio. Mean ± SD values are shown.

a Assessed using data closest to the end of baseline and follow-up.

b eGFR laboratory values were either calculated with the CKD-EPI creatinine equation (2021) for adult patients (aged ≥ 18 years) or reported by Optum (Schwartz formula) for pediatric patients (aged < 18 years).

c Median follow-up was 25.4 mos (IQR: 15.0–39.4) and median time between eGFR values was 22.2 mos (IQR: 10.9–37.3).

d Median follow-up was 23.5 mos (IQR: 12.9–37.1) and median time between proteinuria values was 17.2 mos (IQR: 7.3–30.5).

The use of C3G-related treatments was assessed in a subset of patients with CKD stage progression at 6-, 12-, 24-, and 36-months postindex (Table 5). Supportive therapy use was numerically higher among those who had an indication of progression at about 36 months postindex than among progressors observed at 6 months postindex (68.4% vs. 40.0%). During follow-up, 198 patients (76.2%) had a diagnosis of ≥ 1 cardiovascular condition, including heart failure, myocardial infarction, and stroke or transient ischemic attack, which respectively occurred in 54 (20.8%), 27 (10.4%), and 11 (4.2%) patients with C3G (Supplementary Table S10). Furthermore, acute kidney injury was diagnosed in 82 patients (31.5%).

**Table 5:** Postindex C3G-related treatment in patients with CKD progression at follow-up

C3G-related treatment	Patients with CKD progression at follow-up^a^			
	6 mos^b^ (*n* = 15)	12 mos^c^ (*n* = 25)	24 mos^c^ (*n* = 22)	36 mos^c^ (*n* = 19)
Supportive therapy^d^, *n* (%)	6 (40.0)	11 (44.0)	12 (54.5)	13 (68.4)
ACEi	4 (26.7)	8 (32.0)	7 (31.8)	7 (36.8)
ARBs	3 (20.0)	4 (16.0)	7 (31.8)	7 (36.8)
Immunosuppressive therapy, *n* (%)	5 (33.3)	12 (48.0)	6 (27.3)	7 (36.8)
Corticosteroids (oral/i.v.)	4 (26.7)	10 (40.0)	4 (18.2)	6 (31.6)
Other immunosuppressive agents	3 (20.0)	4 (16.0)	3 (13.6)	3 (15.8)
Mycophenolate mofetil	2 (13.3)	2 (8.0)	2 (9.1)	0
Tacrolimus	2 (13.3)	2 (8.0)	2 (9.1)	1 (5.3)
Eculizumab, *n* (%)	0	0	1 (4.5)	0

ACEi, angiotensin-converting enzyme inhibitors; ARB, angiotensin II receptor blocker; C3G, C3 glomerulopathy; CKD, chronic kidney disease.

a Progression was assessed between CKD stage at index and CKD stage at the follow-up time point among patients with a CKD stage 1–4 at index; patients with a lower CKD stage at index than at the follow-up time point were considered progressed; patients with CKD stage 3 unspecified at index and CKD stage 4 or 5 at the follow-up time point were considered progressed.

b 6 mos from index ± 1 mo.

c 12, 24, or 36 months from index ± 3 mos.

d Supportive therapy is defined as treatment with either ACEi or ARBs.

### Sensitivity Analysis

The results were generally consistent when limited to the sample of patients with ≥ 2 C3G diagnosis codes on distinct dates (Supplementary Sensitivity Analysis).

## Discussion

This is the first real-world, US multicenter analysis to describe the demographic and clinical characteristics of patients with C3G and estimate the timing of CKD stage progression.

In the current analysis, 59.0% of patients with C3G progressed to a higher CKD stage, and 26.0% progressed to CKD stage 5 or kidney failure over a median follow-up of 2.1 years; however, the follow-up time was relatively short, which could contribute to these findings. Relative to nonprogressors, patients who progressed to a higher CKD stage during follow-up had poor kidney function at index date. During baseline, CKD stage progressors had high rates of comorbidities and more frequently received hemodialysis and supportive therapy. The few published C3G cohort studies have found that 11.5% to 40% of patients with C3G progressed to CKD stage 5 or kidney failure across 23 to 72 months.^2^^,^^10^^,^^19^^,^^23^ Other reported factors associated with the risk of kidney disease progression in patients with C3G include proteinuria, use of angiotensin-converting enzyme inhibitors or angiotensin II receptor blockers, age, and dense deposit disease.^5^^,^^10^^,^^19^ At index, most patients in this analysis presented with a proteinuria level ≥ 1 g/g (54.4%) and hematuria (67.4%), mean (SD) eGFR was 61.6 (37.5) ml/min per 1.73 m^2^, and 56.6% of the patients who had CKD stage assessed had CKD stage ≥ 3 at index. Similar laboratory findings have been reported in other C3G cohort studies^2^^,^^5^^,^^10^^,^^19^^,^^24^; however, in the present analysis, a greater proportion of patients had decreased kidney function at index compared with previous studies (∼29.1%–48.5% of patients presenting with CKD stage ≥ 3 at baseline).^5^^,^^19^

In this study, the patient population was stratified by several factors, including C3 level at index and progression status. At baseline, patients with normal C3 levels (63.7%) tended to have more C3G-related comorbidities. Though CKD stage ≥ 3 was more prevalent in patients with normal C3 compared with those with decreased C3, there was minimal difference in both the median time to and the likelihood of CKD stage progression between these groups. However, in the sensitivity analysis sample, patients with decreased C3 levels were more likely to have disease progression to a higher CKD stage than those with normal C3 levels. The wide CI in the sensitivity analysis is likely driven by the small sample size. Patients classified as disease progressors had significantly higher mean CCI scores and more C3G-related comorbidities compared with patients without disease progression. In addition, the use of cardiovascular-related treatments, supportive therapies, and corticosteroids was significantly higher in patients with CKD stage progression compared with non-progressors.

One option for patients with C3G and kidney failure is kidney transplant. However, AP abnormalities frequently remain posttransplantation and contribute to C3G recurrence in a majority of patients; transplants therefore have limited success.^7^^,^^9^^,^^11^^,^^28^^,^^29^ In a study of 19 patients with C3G who underwent kidney transplantation, 16 (84.2%) had recurrent disease and 9 (47.4%) had graft failure.^11^ Similar results were reported in a study of 21 patients with the C3 glomerulonephritis subtype of C3G; following kidney transplantation, 14 (66.7%) had recurrent disease, half of whom experienced graft failure.^29^ These studies highlight a need for novel therapies to prevent C3G recurrence.^11^^,^^30^ In our analysis, 27 patients (10.4%) had received a prior kidney transplant at index and were therefore categorized as having posttransplant recurrent C3G. Although patients with posttransplant recurrent C3G were more likely to receive immunosuppressant agents, it cannot be determined whether the use was for prevention of allograft rejection or to treat C3G recurrence. Patients with posttransplant recurrent C3G had a shorter median time to CKD stage progression (0.5 months) than those with C3G in the native kidney (17.3 months). Patients with posttransplant recurrent C3G also tended to have CKD stage ≥ 3 at index (87.0%), poor kidney function (eGFR ml/min per 1.73 m^2^ mean [SD]: 37.4 [25.1]), and high rates of comorbidities (hypertension, 92.6%). Consequently, patients with posttransplant recurrence represent a population at higher risk of disease progression than those with C3G in the native kidney.

Current treatment options for C3G, recommended by the Kidney Disease: Improving Global Outcomes guidelines, focus on supportive care and immunosuppression, and are based on expert opinion rather than robust clinical trial data.^12^ In our analysis, the proportion of patients receiving supportive therapies was numerically higher among those who had an indication of progression at about 36 months postindex (68.4%) relative to those who progressed at 6 months postindex (40.0%). Despite receiving treatment, disease still progressed, highlighting the unmet need for targeted therapies. Until 2025, there were no treatments that targeted the underlying pathogenesis in C3G.^3^ Iptacopan received US Food and Drug Administration approval in March 2025 for adult patients with C3G to reduce proteinuria, making it the first treatment approved for this condition.^13^ In addition, pegcetacoplan, a C3 and C3b inhibitor, received US Food and Drug Administration approval for the treatment of C3G and primary immune complex membranoproliferative glomerulonephritis in July 2025.^15^ Furthermore, several agents targeting different parts of the complement system are under investigation.^31^, ^32^, ^33^, ^34^, ^35^, ^36^, ^37^ Taking these updates into account, the therapeutic landscape is promising, offering targeted therapeutic options for patients with C3G.

The main strength of this analysis is the population size. This is the largest cohort of US patients with C3G reported to date, with patients from multiple centers, ensuring a diverse and representative population. In addition, the results were generally consistent when limiting to the sample of patients with ≥ 2 C3G diagnosis codes on distinct dates. However, our analysis has several limitations. Because definitive diagnosis of C3G requires a kidney biopsy,^1^ the index was initially defined as the date of first kidney biopsy prior to a C3G diagnosis code. However, there were substantial missing kidney biopsy data, with only 20.4% of patients having a record of kidney biopsy; therefore, patients were identified based on C3G diagnostic codes. In addition, C3G-specific ICD-10-CM codes are recent and therefore limit the ability to identify the condition in real-world data sources. Separately, a proportion could have been identified when C3G was diagnosed as membranoproliferative glomerulonephritis type II before the 2013 consensus report that defined C3G.^38^^,^^39^ Therefore, we may have captured prevalent patients as well as incident patients. Existing membranoproliferative glomerulonephritis ICD codes are nonspecific and may include non-C3G patients, so were not used for patient identification,^40^ which may have led to under detection. Furthermore, patients with infection or endocarditis-associated C3G could have been misclassified. Disease progression was assessed based on CKD stage; however, CKD stage was derived using a combination of diagnosis codes, procedure codes, and eGFR values, and therefore may not reflect the actual CKD stage for each patient. Moreover, the assumption that patients who received a kidney transplant before C3G diagnosis had recurrent C3G may not be accurate. Patients were required to have continuous clinical activity for ≥ 12 months before and ≥ 6 months after index, which may have led to the underestimation of the proportion of patients with select clinical events, such as progression. Finally, there were substantial missing laboratory data, thereby limiting some objectives. This meant we were unable to assess for more specific laboratory measures of interest, for example, presence of nephritic factors.

In this analysis of patients with C3G from a real-world US cohort, we identified a population with multiple comorbidities and advanced kidney disease around the time of C3G diagnosis. Although this cohort comprised the largest population of US patients with C3G analyzed to date, the limitations raised regarding missing biopsy and laboratory data suggest the value and need for US-based registries that include biospecimens, histology data, genetics, and patient-reported outcomes in C3G. Despite supportive care, there was progression of CKD among the patients in our cohort, highlighting the need to use novel treatments to improve outcomes in patients with C3G.

## Disclosure

BCN and JN are employees of Novartis Pharmaceuticals Corporation. CA was an employee of Novartis Pharmaceuticals Corporation at the time of the study, and is a current employee of Bayer US LLC. IP, MLE, AA, and JS are employees of Analysis Group. PAC has consultancy agreements with Chinook Therapeutics, Novartis Pharmaceuticals Corporation, Otsuka Pharmaceutical, and Takeda, and has received research funding from Calliditas Therapeutics, Novartis Pharmaceuticals Corporation, and Travere Therapeutics.

## Supplementary Material

Supplementary File (PDF) **Supplementary Sensitivity Analysis. Figure S1.** Study design. **Table S1.** Diagnosis codes for C3G. **Table S2.** Diagnosis codes for CKD staging. **Table S3.** Comorbidities for the overall population and stratified by kidney transplant status, C3 level, and progression status. **Table S4.** Diagnosis codes for comorbidities in the CCI. **Table S5.** Diagnosis codes for C3G-related comorbidities. **Table S6.** C3G-related treatments. **Table S7.** CKD stage progression over time. **Table S8.** Relationship between kidney transplant status and baseline C3 level and progression to a higher CKD stage/stage 5. **Table S9.** Postindex laboratory values in patients with CKD progression at follow-up. **Table S10.** Cardiovascular conditions, acute kidney injuries, and infections during follow-up in patients with C3G. STROBE Checklist: cohort studies.

## Footnotes

Supplementary File (PDF)

**Supplementary Sensitivity Analysis**
**Figure S1.** Study design. **Table S1.** Diagnosis codes for C3G. **Table S2.** Diagnosis codes for CKD staging. **Table S3.** Comorbidities for the overall population and stratified by kidney transplant status, C3 level, and progression status. **Table S4.** Diagnosis codes for comorbidities in the CCI. **Table S5.** Diagnosis codes for C3G-related comorbidities. **Table S6.** C3G-related treatments. **Table S7.** CKD stage progression over time. **Table S8.** Relationship between kidney transplant status and baseline C3 level and progression to a higher CKD stage/stage 5. **Table S9.** Postindex laboratory values in patients with CKD progression at follow-up. **Table S10.** Cardiovascular conditions, acute kidney injuries, and infections during follow-up in patients with C3G. STROBE Checklist: cohort studies.
