# Supplementary material for: Real-World Analysis of Patients With C3 Glomerulopathy in the United States
Source: Kidney Int Rep. 2026 Jan 14;11(3):103773. doi: 10.1016/j.ekir.2026.103773 (PMC12914277; doi:10.1016/j.ekir.2026.103773)
Supplement: Supplementary File (PDF) — Supplementary Sensitivity Analysis. Figure S1. Study design. Table S1. Diagnosis codes for C3G. Table S2. Diagnosis codes for CKD staging. Table S3. Comorbidities for the overall population and stratified by kidney transplant status, C3 level, and progression status. Table S4. Diagnosis codes for comorbidities in the CCI. Table S5. Diagnosis codes for C3G-related comorbidities. Table S6. C3G-related treatments. Table S7. CKD stage progression over time. Table S8. Relationship between kidney transplant status and baseline C3 level and progression to a higher CKD stage/stage 5. Table S9. Postindex laboratory values in patients with CKD progression at follow-up. Table S10. Cardiovascular conditions, acute kidney injuries, and infections during follow-up in patients with C3G. STROBE Checklist: cohort studies. [file mmc1.pdf]

SUPPLEMENTARY MATERIAL

Supplementary Sensitivity Analysis

Supplementary Table S1. Diagnosis codes for C3G

Supplementary Table S2. Diagnosis codes for CKD staging

Supplementary Table S3. Comorbidities for the overall population and stratified by kidney transplant, C3 level, and progression status

Supplementary Table S4. Diagnosis codes for comorbidities in the CCI

Supplementary Table S5. Diagnosis codes for C3G-related comorbidities

Supplementary Table S6. C3G-related treatments

Supplementary Table S7. CKD stage progression over time

Supplementary Table S8. Relationship between transplant status and baseline C3 level and progression to a higher CKD stage/stage 5

Supplementary Table S9. Post-index laboratory values in patients with CKD progression at follow-up

Supplementary Table S10. Cardiovascular conditions, acute kidney injuries, and infections during follow-up in patients with C3G

Supplementary Figure S1. Study design

STROBE Checklist: cohort studies

Sensitivity Analysis

Baseline Patient Demographics, Clinical Characteristics, and Treatments

A total of 139/260 (53.5%) patients in the full study sample met the requirement of having ≥2 C3 glomerulopathy (C3G) diagnosis codes on distinct dates and were included in the sensitivity analysis sample. These patients had relatively similar demographic and clinical characteristics compared with the main study sample; categorical variables had differences of ~5% or less. In the sensitivity analysis sample, mean (SD) age at index was 45.8 (20.6) years (~2 years younger than the full study sample), 79.1% of patients were White, and 49.6% were female. Of the 114 patients with available chronic kidney disease (CKD) stage data at index, 68 (59.6%) had CKD stage ≥3. Based on data closest to index, 30/51 (58.8%) patients with available data had normal C3 levels. Of patients with proteinuria values at baseline, 34/67 (50.7%) had a proteinuria level ≥1 g/g. Mean (SD) number of red blood cells per high power field was 29.6 (48.1) (among n = 53, 38.1%), and mean (SD) estimated glomerular filtration rate (eGFR) was 59.6 (36.9) ml/min/1.73 m^2 (among n = 118, 84.9%). The mean (SD) Charlson Comorbidity Index score was 2.1 (2.4), hypertension was the most common C3G-related comorbidity (61.9%), and the most common C3G-related treatments prior to index were cardiovascular-related (60.4%) and supportive therapies (59.0%).

CKD Stage Progression

Results related to CKD stage progression outcomes also remained similar in the sensitivity analysis sample. Among those assessed for CKD stage progression (n = 94), 30 (31.9%) progressed to CKD stage 5/kidney failure during follow-up compared with 26.0% in the full study sample. At index date, relative to non-progressors, patients who had disease progression were more likely to have CKD stage ≥3 (63.8% vs 30.6%, $P < 0.05$). Median time to disease progression was 15.5 months (95% confidence interval [CI]: 10.5–22.3) compared with 14.9 months (95% CI: 10–21.9) in the full sample. Median time to CKD stage 5/kidney failure was not reached, which was similar to the full study sample.

Of the patients assessed for CKD stage progression (n = 94), 7 (7.4%) had post-transplant recurrent C3G, of whom 6 (85.7%) experienced disease progression during the observation period compared with 84.6% in the full study sample. Among patients with native kidneys who were assessed for CKD stage progression (n = 87), 52 (59.8%) showed disease progression compared with 56.9% in the full sample. Median time to disease progression was significantly shorter in patients with post-transplant recurrent C3G (0.1 months [95% CI: 0.0–not estimable (NE)]) compared with patients with C3G in the native kidney (17.5 months [95% CI: 12.3–25.5], P < 0.001). Unlike the full study sample, in the sensitivity analysis there was no significant difference in the likelihood of disease progression to a higher CKD stage between patients with post-transplant recurrent C3G and those with C3G in the native kidney, albeit with a wide CI reflecting the limited sample size (hazard ratio [HR]: 2.40 [95% CI: 0.72–8.03], P = 0.16).

Of the 35 patients assessed for CKD stage progression who had a C3 level at index date, 22 (62.9%) had normal C3 levels and 13 (37.1%) had decreased C3 levels. Disease progression occurred in 16 (72.7%) and 9 (69.2%) patients with normal and decreased C3 levels, respectively. Median time to disease progression among patients with normal and decreased C3 levels was similar at 9.4 months (95% CI: 2.3–NE) and 9.4 months (95% CI: 0.2–NE), respectively; these results were also similar to those of the full study sample: 10.0 months (95% CI: 4.3–NE) and 9.4 months (95% CI: 1.0–NE), respectively. Patients with decreased C3 levels were 4 times more likely to have disease progression to a higher CKD stage than those with normal C3 levels (HR: 4.05 [95% CI: 1.23–13.29], P < 0.05). This differs from the main study sample, where there was no significant difference in progression risk to a higher CKD stage between patients with decreased C3 levels and those with normal C3 levels (HR: 1.25 [95% CI: 0.58–2.70], P = 0.56).

Post-Index Clinical Characteristics

Median (interquartile range [IQR]) annual eGFR slope in the sensitivity analysis sample, calculated for each patient (n = 96) as the change in eGFR per year from baseline to the end

of follow-up, was –0.8 (–7.6, 5.1) ml/min/1.73 m^2/year, which was similar to the full study sample (–0.9 [–7.5, 3.8] ml/min/1.73 m^2/year). Median (IQR) monthly proteinuria slope in the sensitivity analysis sample, calculated for each patient (n = 50) as the change in proteinuria from baseline to the end of follow-up, was 0.0 (0.0–0.0) g/g/month; this was similar to the main study sample (0.0 [–0.1, 0.0] g/g/month).

Supplementary Tables

Supplementary Table S1. Diagnosis codes for C3G

Code type	Diagnosis code	Condition
ICD-10-CM	N00.6	Acute nephritic syndrome with DDD
	N01.6	Rapidly progressive nephritic syndrome with DDD
	N02.6	Recurrent and persistent hematuria with DDD
	N03.6	Chronic nephritic syndrome with DDD
	N04.6	Nephrotic syndrome with DDD
	N05.6	Unspecified nephritic syndrome with DDD
	N06.6	Isolated proteinuria with DDD
	N07.6	Hereditary nephropathy not elsewhere classified with DDD
	N00.A	Acute nephritic syndrome with C3GN
	N01.A	Rapidly progressive nephritic syndrome with C3GN
	N02.A	Recurrent and persistent hematuria with C3GN
	N03.A	Chronic nephritic syndrome with C3GN
	N04.A	Nephrotic syndrome with C3GN
	N05.A	Unspecified nephritic syndrome with C3GN
	N06.A	Isolated proteinuria with C3GN
	N07.A	Hereditary nephropathy not elsewhere classified with C3GN
SNOMED CT	722758004	C3 glomerulopathy (disorder)
	197724001	Isolated proteinuria with specified morphological lesion (finding)
	722761003	C3GN (disorder)
	59479006	Mesangiocapillary glomerulonephritis, type II

	722760002	DDD
	197599000	Nephrotic syndrome, DDD (disorder)
	197687001	Acute nephritic syndrome, DDD
	197696001	Rapidly progressive nephritic syndrome, DDD
	197714009	Chronic nephritic syndrome, DDD

C3, complement component 3; C3GN, complement 3 glomerulonephritis; DDD, dense deposit disease; ICD-10-CM, International Classification of Diseases, 10th Revision, Clinical Modification; SNOMED CT, Systemized Nomenclature of Medicine Clinical Terms.

Supplementary Table S2. Diagnosis codes for CKD staging

**CKD stage**	**ICD-9-CM codes**	**ICD-10-CM codes**
Stage 1	585.1	N18.1
Stage 2	585.2	N18.2
Stage 3 unspecified	585.3	N18.3 N18.30
Stage 3a		N18.31
Stage 3b		N18.32
Stage 4	585.4	N18.4
Stage 5/kidney failure	403.01 404.13<br>403.11 404.92<br>403.91 404.93<br>404.02 585.5<br>404.03 585.6<br>404.12	N18.5<br>N18.6<br>I12.0<br>I13.11<br>I13.2

CKD, chronic kidney disease; eGFR, estimated glomerular filtration rate; ICD-9-CM, International Classification of Diseases, 9th Revision, Clinical Modification; ICD-10-CM, International Classification of Diseases, 10th Revision, Clinical Modification.

CKD stage was defined using the eGFR laboratory values closest the index date. If eGFR data were not available in the specified time (index date ± 1 month), diagnosis data within the time was considered and CKD stage was defined using the CKD diagnosis codes closest to index. If a patient had a procedure code for dialysis within the specified time (index date ± 1 month), their CKD stage for that time was defined as CKD stage 5/kidney failure, regardless of eGFR laboratory values and diagnosis data availability.

Supplementary Table S3. Comorbidities for the overall population and stratified by kidney transplant status, C3 level, and progression status

		Kidney status at index (*n* = 260)			Status by C3 level[a] (*n* = 91)			Patients with CKD stage assessed during follow-up[b] (*n* = 173)		
**Characteristic**	**Overall (*N* = 260)**	**C3G in the native kidney (*n* = 233)**	**Post-transplant recurrent C3G (*n* = 27)**	***P* value**	**Decreased (*n* = 33)**	**Normal (*n* = 58)**	***P* value**	**Non-progressors (*n* = 71)**	**CKD stage progressors (*n* = 102)**	***P* value**
**CCI score**										
Mean ± SD	2.1 ± 2.5	2.0 ± 2.4	3.3 ± 2.8	< 0.05*	3.0 ± 3.3	2.5 ± 2.2	0.35	1.8 ± 2.4	2.7 ± 2.6	< 0.05*
**Comorbidities included in the CCI,[c] *n* (%)**										
Kidney disease[d]	151 (58.1)	124 (53.2)	27 (100.0)	< 0.001*	28 (84.8)	46 (79.3)	0.71	38 (53.5)	67 (65.7)	0.15
Chronic pulmonary disease	64 (24.6)	56 (24.0)	8 (29.6)	0.69	7 (21.2)	16 (27.6)	0.67	16 (22.5)	30 (29.4)	0.41
Diabetes without chronic complication	49 (18.8)	40 (17.2)	9 (33.3)	0.08	3 (9.1)	13 (22.4)	0.15	10 (14.1)	30 (29.4)	< 0.05*
Congestive heart failure	48 (18.5)	39 (16.7)	9 (33.3)	0.07	9 (27.3)	17 (29.3)	1.00	10 (14.1)	22 (21.6)	0.29

Malignancy	31 (11.9)	26 (11.2)	5 (18.5)	0.42	3 (9.1)	7 (12.1)	0.74	8 (11.3)	14 (13.7)	0.81
Peripheral vascular disease	32 (12.3)	25 (10.7)	7 (25.9)	< 0.05*	3 (9.1)	7 (12.1)	0.74	9 (12.7)	17 (16.7)	0.61
Cerebrovascular disease	35 (13.5)	29 (12.4)	6 (22.2)	0.27	6 (18.2)	13 (22.4)	0.83	9 (12.7)	20 (19.6)	0.32
Mild liver disease	33 (12.7)	28 (12.0)	5 (18.5)	0.51	9 (27.3)	9 (15.5)	0.28	5 (7.0)	20 (19.6)	< 0.05*
Diabetes with chronic complication	31 (11.9)	26 (11.2)	5 (18.5)	0.42	2 (6.1)	9 (15.5)	0.32	3 (4.2)	22 (21.6)	< 0.01*
**C3G-related comorbidities, *n* (%)**									
Hypertension	163 (62.7)	138 (59.2)	25 (92.6)	< 0.001*	20 (60.6)	46 (79.3)	0.09	38 (53.5)	81 (79.4)	< 0.001*
Fatigue/tiredness	84 (32.3)	70 (30.0)	14 (51.9)	< 0.05*	8 (24.2)	30 (51.7)	< 0.05*	22 (31.0)	36 (35.3)	0.67
Edema	66 (25.4)	59 (25.3)	7 (25.9)	1.00	7 (21.2)	24 (41.4)	0.09	14 (19.7)	36 (35.3)	< 0.05*
Pain	52 (20.0)	44 (18.9)	8 (29.6)	0.29	5 (15.2)	13 (22.4)	0.57	8 (11.3)	31 (30.4)	< 0.01*

C3, complement component 3; C3G, C3 glomerulopathy; CCI, Charlson Comorbidity Index; CKD, chronic kidney disease; ICD-9-CM, International Classification of Diseases, 9th Revision, Clinical Modification; ICD-10-CM, International Classification of Diseases, 10th Revision, Clinical Modification.

[a]Assessed using data closest to index.

[b]Patients with a lower CKD stage at index than at the follow-up timepoint were considered progressed.

[c]≥10% in the overall population.

[d]Kidney disease includes select kidney conditions as defined per the CCI, based on the presence of an ICD-9-CM or ICD-10-CM code.

*Indicates $P < 0.05$.

Supplementary Table S4. Diagnosis codes for comorbidities in the CCI

Condition	ICD-9-CM codes		ICD-10-CM codes	
Myocardial infarction	410<br>412		I21<br>I22	I25.2
Congestive heart failure	398.91<br>402.01<br>402.11<br>402.91<br>404.01<br>404.03	404.11<br>404.13<br>404.91<br>404.93<br>425.4–425.9<br>428	I09.9<br>I11.0<br>I13.0<br>I13.2<br>I25.5<br>I42.0	I42.5–I42.9<br>I43<br>I50<br>P29.0
Peripheral vascular disease	093.0<br>437.3<br>440<br>441<br>443.1–443.9<br>447.1<br>557.1	557.9<br>V43.4	I70<br>I71<br>I73.1<br>I73.8<br>I73.9<br>I77.1<br>I79.0	I79.2<br>K55.1<br>K55.8<br>K55.9<br>Z95.8<br>Z95.9
Cerebrovascular disease	362.34<br>430-438		G45<br>G46	H34.0<br>I60–I69
Dementia	290<br>294.1	331.2	F00–F03<br>F051	G30<br>G31.1
Chronic pulmonary disease	416.8<br>416.9<br>490–505<br>506.4	508.1<br>508.8	I27.8<br>I27.9<br>J40–J47<br>J60–J67	J68.4<br>J70.1<br>J70.3
Rheumatic disease	446.5<br>710.0–710.4	725	M05<br>M06	M35.1<br>M35.3

	714.0–714.2<br>714.8		M31.5<br>M32–M34	M36.0
Peptic ulcer disease	531–534		K25–K28	
Liver disease, mild	070.22<br>070.23<br>070.32<br>070.33<br>070.44<br>070.54<br>070.6<br>070.9	570<br>571<br>573.3<br>573.4<br>573.8<br>573.9<br>V42.7	B18<br>K70.0–K70.3<br>K70.9<br>K71.3–K71.5<br>K71.7<br>K73<br>K74<br>K76.0	K76.2–K76.4<br>K76.8<br>K76.9<br>Z94.4
Diabetes without chronic complications	250.0–250.3<br>250.8<br>250.9		E10.0<br>E10.1<br>E10.6<br>E10.8<br>E10.9<br>E11.0<br>E11.1<br>E11.6<br>E11.8<br>E11.9<br>E12.0<br>E12.1<br>E12.6	E12.8<br>E12.9<br>E13.0<br>E13.1<br>E13.6<br>E13.8<br>E13.9<br>E14.0<br>E14.1<br>E14.6<br>E14.8<br>E14.9

Diabetes with chronic complications	250.4–250.7		E10.2–E10.5<br>E10.7<br>E11.2–E11.5<br>E11.7<br>E12.2–E12.5	E12.7<br>E13.2–E13.5<br>E13.7<br>E14.2–E14.5<br>E14.7
Hemiplegia or paraplegia	334.1<br>342<br>343<br>344.0–344.6	344.9	G04.1<br>G11.4<br>G80.1<br>G80.2	G81<br>G82<br>G83–G83.4<br>G83.9
Renal disease	403.01<br>403.11<br>403.91<br>404.02<br>404.03<br>404.12<br>404.13<br>404.92<br>404.93	582<br>583.0–583.7<br>585<br>586<br>588.0<br>V42.0<br>V45.1<br>V56	I12.0<br>I13.1<br>N03.2–N03.7<br>N05.2–N05.7<br>N18<br>N19<br>N25.0<br>Z49.0–Z49.2<br>Z94.0	Z99.2
Any malignancy[a]	140–172<br>174–195.8<br>200–208<br>238.6		C00–C26<br>C30–C34<br>C37–C41<br>C43<br>C45–C58	C60–C76<br>C81–C85<br>C88<br>C90–C97
Liver disease, moderate or severe	456.0–456.2<br>572.2–572.8		I85.0<br>I85.9<br>I86.4<br>I98.2	K72.1<br>K72.9<br>K76.5– K76.7

		K70.4<br>K71.1
Metastatic solid tumor	196–199	C77–C80
AIDS/HIV	042–044	B20–B22 B24

AIDS, acquired immunodeficiency syndrome; CCI, Charlson Comorbidity Index; HIV, human immunodeficiency virus; ICD-9-CM, International Classification of Diseases, 9th Revision, Clinical Modification; ICD-10-CM, International Classification of Diseases, 10th Revision, Clinical Modification.

[a]Including leukemia and lymphoma, except for malignant neoplasm of skin.

Supplementary Table S5. Diagnosis codes for C3G-related comorbidities

Condition	ICD-9-CM codes		ICD-10-CM codes	
Edema	782.3		R60.0<br>R60.1	R60.9
Fatigue/tiredness	780.7		R53	
Pain	338	780.96	G89	R52
Hypertension	401–405		I10– I13	I15
Kidney transplant	V42.0		T86.10–T86.13<br>T86.19	Z94.0<br>Z48.22

C3G, C3 glomerulopathy; ICD-9-CM, International Classification of Diseases, 9th Revision, Clinical Modification; ICD-10-CM, International Classification of Diseases, 10th Revision, Clinical Modification.

Supplementary Table S6. C3G-related treatments

**Drug class**	**Medication**
Monoclonal antibodies	Eculizumab
ACE inhibitors	Benazepril<br>Captopril<br>Enalapril<br>Fosinopril<br>Lisinopril<br>Moexipril<br>Perindopril<br>Quinapril<br>Ramipril<br>Trandolapril
ARBs	Azilsartan medoxomil<br>Candesartan<br>Eprosartan<br>Irbesartan<br>Losartan<br>Olmesartan<br>Telmisartan<br>Valsartan
Immunosuppressive agents	Alemtuzumab<br>Azathioprine<br>Basiliximab<br>Belatacept<br>Cyclophosphamide<br>Cyclosporin

	Everolimus<br>Methotrexate<br>Mycophenolate mofetil<br>Mycophenolate sodium<br>Rabbit antithymocyte globulin<br>Rituximab (and biosimilars)<br>Sirolimus<br>Tacrolimus
Oral corticosteroids	Cortisone<br>Dexamethasone<br>Hydrocortisone<br>Methylprednisolone<br>Prednisolone<br>Prednisone
Cardiovascular-related treatments	Beta blockers<br>Diuretics<br>Mineralocorticoid receptor antagonists<br>Statins

ACE, angiotensin-converting enzyme; ARB, angiotensin II receptor blocker; C3G, C3 glomerulopathy.

Supplementary Table S7. CKD stage progression over time

		Kidney transplant status at index (*n* = 260)		Status by C3 level (*n* = 91)	
	**Overall (*N* = 260)**	**Post-transplant recurrent C3G (*n* = 27)**	**C3G in the native kidney (*n* = 233)**	**Decreased (*n* = 33)**	**Normal (*n* = 58)**
**CKD stage progression at 6 months,[a] *n* (%)**					
Progression assessed[b]	80 (30.8)	8 (29.6)	72 (30.9)	14 (42.4)	28 (48.3)
Progressed CKD stage	15 (18.8)	4 (50.0)	11 (15.3)	2 (14.3)	3 (10.7)
Progressed to CKD stage 5/kidney failure	5 (6.3)	1 (12.5)	4 (5.6)	0	0
**CKD stage progression at 12 months,[c] *n* (%)**					
Progression assessed[b]	91 (35.0)	5 (18.5)	86 (36.9)	11 (33.3)	27 (46.6)
Progressed CKD stage	25 (27.5)	3 (60.0)	22 (25.6)	2 (18.2)	8 (29.6)
Progressed to CKD stage 5/kidney failure	10 (11.0)	1 (20.0)	9 (10.5)	1 (9.1)	3 (11.1)
**CKD stage progression at 24 months,[c] *n* (%)**					
Progression assessed[b]	60 (23.1)	2 (7.4)	58 (24.9)	7 (21.2)	16 (27.6)

Progressed CKD stage	22 (36.7)	2 (100.0)	20 (34.5)	2 (28.6)	7 (43.8)
Progressed to CKD stage 5/kidney failure	10 (16.7)	0	10 (17.2)	2 (28.6)	2 (12.5)
**CKD stage progression at 36 months,[c] *n* (%)**					
Progression assessed[b]	40 (15.4)	1 (3.7)	39 (16.7)	6 (18.2)	8 (13.8)
Progressed CKD stage	19 (47.5)	1 (100.0)	18 (46.2)	2 (33.3)	3 (37.5)
Progressed to CKD stage 5/kidney failure	9 (22.5)	0	9 (23.1)	2 (33.3)	1 (12.5)

C3, complement component 3; C3G, C3 glomerulopathy; CKD, chronic kidney disease.

[a]6months from index ± 1 month.

[b]Progression assessment required CKD stage at index and follow-up timepoint among patients with CKD stage 1–4.

[c]12, 24, or 36 months from index ± 3 months.

Supplementary Table S8. Relationship between kidney transplant status and baseline C3 level and progression to a higher CKD stage/stage 5

	**HR**	**95% CI**	***P* value**
**Progressed to higher CKD stage**[a]			
Transplant status at index[b,c]	4.01	1.70–9.46	< 0.01
Decreased baseline C3 level[d,e]	1.25	0.58–2.70	0.56
**Progressed to CKD stage 5**			
Transplant status at index[b,c]	2.04	0.64–6.47	0.23
Decreased baseline C3 level[d,e]	1.53	0.47–4.94	0.48

BMI, body mass index; C3, complement 3; CCI, Charlson Comorbidity Index; CKD, chronic kidney disease; CI, confidence interval; HR, hazard ratio.

[a]Patients with a higher CKD stage after index than their CKD stage at index were considered progressed.

[b]Index date transplant status was assessed based on whether a patient had a kidney transplant during baseline.

[c]The relationships between transplant status at index and progression to a higher CKD stage and to CKD stage 5 were examined with a multivariable Cox proportional hazards model on 173 patients, and adjusted for age at index, sex, CKD stage at index, baseline CCI score, baseline supportive therapy, and baseline immunosuppressive agents.

[d]Baseline C3 level was assessed using the data closest to index.

[e]The relationships between baseline C3 level and progression to higher CKD stage and to CKD stage 5 were examined with a multivariable Cox proportional hazards model on 62 patients, and adjusted for age at index date, sex, CKD stage at index, baseline BMI, and baseline supportive therapy.

Supplementary Table S9. Post-index laboratory values in patients with CKD progression at follow-up

Laboratory assessments	Patients with CKD progression at post-index follow-up[a]			
	**6 months[b] (*n* = 15)**	**12 months[c] (*n* = 25)**	**24 months[c] (*n* = 22)**	**36 months[c] (*n* = 19)**
**eGFR (ml/min/1.73 m^2),[d] *n* (%)**				
eGFR assessed	13 (86.7)	22 (88.0)	22 (100.0)	17 (89.5)
Mean ± SD	39.0 ± 17.0	38.7 ± 25.9	29.3 ± 20.9	37.3 ± 29.0
**Proteinuria (g/g), *n* (%)**				
Proteinuria assessed	6 (40.0)	11 (44.0)	5 (22.7)	2 (10.5)
UPCR, mean ± SD	2.8 ± 4.1	1.8 ± 1.8	4.0 ± 4.4	0.9 ± 0.3

CKD, chronic kidney disease; CKD-EPI, Chronic Kidney Disease Epidemiology Collaboration; eGFR, estimated glomerular filtration rate; UPCR, urine total protein:creatinine ratio.

[a]Progression was assessed between CKD stage at index and CKD stage at the follow-up timepoint among patients with a CKD stage 1–4 at index. Patients with a lower CKD stage at index than at the follow-up timepoint were considered progressed. Patients with CKD stage 3 unspecified at index and CKD stage 4 or 5 at the follow-up timepoint were considered progressed.

[b]6 months from index ± 1 month.

[c]12, 24, or 36 months from index ± 3 months.

[d]eGFR laboratory values were either calculated with the CKD-EPI creatinine equation (2021) for adult patients (aged ≥18 years) or reported by Optum (Schwartz formula) for pediatric patients (aged <18 years.

Supplementary Table S10. Cardiovascular conditions, acute kidney injuries, and infections during follow-up in patients with C3G

	**Overall (*N* = 260)**
**Cardiovascular conditions,[a] *n* (%)**	
Diagnosis	198 (76.2)
Heart failure, *n* (%)	
Diagnosis	54 (20.8)
Hospitalizations	33 (61.1)
Myocardial infarction, *n* (%)	
Diagnosis	27 (10.4)
Hospitalizations	19 (70.4)
Stroke or transient ischemic attack, *n* (%)	
Diagnosis	11 (4.2)
Hospitalizations	6 (54.5)
**Acute kidney injury, *n* (%)**	
Diagnosis	82 (31.5)
Hospitalizations	57 (69.5)
**Infections, *n* (%)**	
Upper and lower respiratory tract infection	74 (28.5)
Skin and soft tissue infection	36 (13.8)
Urinary tract infection	36 (13.8)
Bacterial disease[b]	34 (13.1)
Mycoses	18 (6.9)
Viral hepatitis	9 (3.5)
Endocarditis	6 (2.3)
Herpes zoster (shingles)	5 (1.9)

C3G, C3 glomerulopathy; ICD-9-CM, International Classification of Diseases, 9th Revision, Clinical Modification; ICD-10-CM, International Classification of Diseases, 10th Revision, Clinical Modification.

These are not *de novo* events and instead reflect the number of patients who had any condition during follow-up, regardless of if they had an event during baseline.

[a]ICD-9-CM codes: 393–398, 401–405, 410–414, 420–429, 433.01, 433.11, 433.21, 433.31, 433.81, 433.91, 434.01, 434.11, 434.91, 435.x; ICD-10-CM codes: I05–I09, I10–I16, I20–I25, I30–I52, I63, G45.x.

[b]Excluded acute respiratory tract infections.

Supplementary Figure

Supplementary Figure S1. Study design

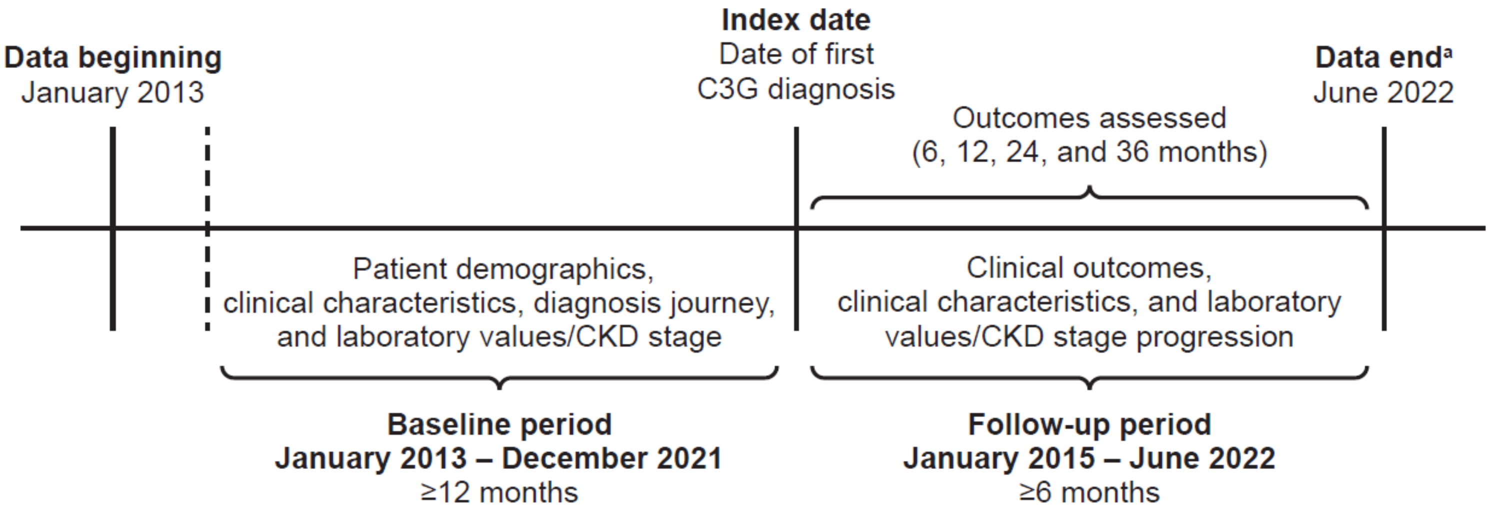

C3G, C3 glomerulopathy; CKD, chronic kidney disease. [a]Or patient death or end of continuous clinical activity, if before data end.

STROBE Checklist: cohort studies

	Item No	Recommendation	Page No
**Title and abstract**	1	(*a*) Indicate the study's design with a commonly used term in the title or the abstract	2
		(*b*) Provide in the abstract an informative and balanced summary of what was done and what was found	2
**Introduction**			
Background/rationale	2	Explain the scientific background and rationale for the investigation being reported	3-4
Objectives	3	State specific objectives, including any prespecified hypotheses	7
**Methods**			
Study design	4	Present key elements of study design early in the paper	4-5
Setting	5	Describe the setting, locations, and relevant dates, including periods of recruitment, exposure, follow-up, and data collection	4-7
Participants	6	(*a*) Give the eligibility criteria, and the sources and methods of selection of participants. Describe methods of follow-up	5-6
		(*b*) For matched studies, give matching criteria and number of exposed and unexposed	n/a
Variables	7	Clearly define all outcomes, exposures, predictors, potential confounders, and effect modifiers. Give diagnostic criteria, if applicable	4-7; Supplementary

			Tables S1, S2, S4, and S5
Data sources/ measurement	8*	For each variable of interest, give sources of data and details of methods of assessment (measurement). Describe comparability of assessment methods if there is more than one group	4-7
Bias	9	Describe any efforts to address potential sources of bias	4-7
Study size	10	Explain how the study size was arrived at	4-5
Quantitative variables	11	Explain how quantitative variables were handled in the analyses. If applicable, describe which groupings were chosen and why	7
Statistical methods	12	(*a*) Describe all statistical methods, including those used to control for confounding	7
		(*b*) Describe any methods used to examine subgroups and interactions	7
		(*c*) Explain how missing data were addressed	n/a
		(*d*) If applicable, explain how loss to follow-up was addressed	n/a
		(*e*) Describe any sensitivity analyses	n/a
**Results**			
Participants	13*	(a) Report numbers of individuals at each stage of study—eg numbers potentially eligible, examined for eligibility, confirmed eligible, included in the study, completing follow-up, and analysed	8, Figure 1
		(b) Give reasons for non-participation at each stage	Figure 1

		(c) Consider use of a flow diagram	Figure 1
Descriptive data	14*	(a) Give characteristics of study participants (eg demographic, clinical, social) and information on exposures and potential confounders	8
		(b) Indicate number of participants with missing data for each variable of interest	8-12
		(c) Summarise follow-up time (eg, average and total amount)	8
Outcome data	15*	Report numbers of outcome events or summary measures over time	8-12

Main results	16	(*a*) Give unadjusted estimates and, if applicable, confounder-adjusted estimates and their precision (eg, 95% confidence interval). Make clear which confounders were adjusted for and why they were included<br>(*b*) Report category boundaries when continuous variables were categorized<br>(*c*) If relevant, consider translating estimates of relative risk into absolute risk for a meaningful time period	8-12
Other analyses	17	Report other analyses done—eg analyses of subgroups and interactions, and sensitivity analyses	8-12
**Discussion**			
Key results	18	Summarise key results with reference to study objectives	13-16

Limitations	19	Discuss limitations of the study, taking into account sources of potential bias or imprecision. Discuss both direction and magnitude of any potential bias	15-16
Interpretation	20	Give a cautious overall interpretation of results considering objectives, limitations, multiplicity of analyses, results from similar studies, and other relevant evidence	13-16
Generalisability	21	Discuss the generalisability (external validity) of the study results	13-16
**Other information**			
Funding	22	Give the source of funding and the role of the funders for the present study and, if applicable, for the original study on which the present article is based	17

*Give information separately for exposed and unexposed groups.

Note: An Explanation and Elaboration article discusses each checklist item and gives methodological background and published examples of transparent reporting. The STROBE checklist is best used in conjunction with this article (freely available on the Web sites of PLoS Medicine at http://www.plosmedicine.org/, Annals of Internal Medicine at http://www.annals.org/, and Epidemiology at http://www.epidem.com/). Information on the STROBE Initiative is available at http://www.strobe-statement.org.
